# Emerging trends and research foci of neuromyelitis optica spectrum disorder: a 20-year bibliometric analysis

**DOI:** 10.3389/fimmu.2023.1177127

**Published:** 2023-06-06

**Authors:** Yue Su, Zhe Ruan, Shicao Li, Zhuyi Li, Ting Chang

**Affiliations:** ^1^ Department of Neurology, Tangdu Hospital, The Fourth Military Medical University, Xi’an, China; ^2^ Department of Pharmacy, Tangdu Hospital, The Fourth Military Medical University, Xi’an, China

**Keywords:** neuromyelitis optica spectrum disorder, NMOSD, bibliometric, Citespace, VOSviewer

## Abstract

**Background:**

Neuromyelitis optica spectrum disorder (NMOSD) is a demyelinating syndrome of the central nervous system. A tremendous amount of literature on NMOSD has been published. This study aimed to perform a bibliometric analysis of the publications on NMOSD and show its hotspots and development trends.

**Methods:**

We used the Web of Science Core Collection as a database and searched the literature published between 2002 and 2022. CiteSpace, VOSviewer, online bibliometric platform, and R-bibliometrix were used to conduct bibliometric analysis and network visualization, including the number of publications, citations, countries/regions, institutions, journals, authors, references, and keywords.

**Results:**

A total of 3,057 publications on NMOSD were published in 198 journals by 200 authors at 200 institutions from 93 countries/regions. The United States published the most literature and made great contributions to this field. The Mayo Clinic was the institution with the largest number of publications. The journal with the most publications was *Multiple Sclerosis and Related Disorders*, and the most co-cited journal was *Neurology*. The author with the most publications was Fujihara, K., while the most frequently co-cited author was Wingerchuk, DM. The current research hotspots may be focused on “efficacy,” “multicenter,” “interleukin-6 receptor blockade,” “safety,” “azathioprine,” “tolerance,” and “adult”.

**Conclusion:**

This study was the first bibliometric analysis of publications on the NMOSD field, visualizing its bibliometric characteristics and gaining insight into the direction, hotspots, and development of global NMOSD research, which may provide helpful information for researchers. Future research hotspots might be conducting randomized controlled trials on targeted immunotherapy in the NMOSD field.

## Introduction

Neuromyelitis optica spectrum disorder (NMOSD) is a central nervous system (CNS) autoimmune inflammatory demyelinating disease characterized by recurrent episodes of acute optic neuritis and transverse myelitis (1). The global annual incidence of NMOSD was estimated at 0.037-0.37 per 100,000 person-year, and the global prevalence was 0.7-10.0 per 100,000 persons (2). For almost a century, neuromyelitis optica (NMO) was considered a variant of multiple sclerosis (MS) that spared the brain (3). It is considered a monophasic disease with bilateral optic neuritis and transverse myelitis, but relapsing cases have been reported in the 20th century (4). A breakthrough was the discovery that the majority of patients with NMO had detectable serum antibodies to aquaporin 4 (AQP4) that were not detected in MS. It is highly specific for clinical diagnosis and has proven NMO to be a distinct disease with a chronic, relapsing course (5, 6). More than 80% of patients with NMOSD have AQP4-IgG autoantibodies (7, 8). NMOSD manifests clinically with six core symptoms, which were classified by their location: optic neuritis, transverse myelitis, area postrema syndrome, brain-stem syndrome, cerebral syndrome, and symptomatic narcolepsy or acute diencephalic clinical syndrome. In 2015, the international NMO diagnostic panel revised a new international diagnostic standard for NMOSD (9). The onset of NMOSD typically reaches its worst within a few days, often leaving a moderate to severe and permanent disability (10). Current treatment options for NMOSD include acute relapse treatment with intravenous glucocorticoids and preventive immunotherapy with a variety of non-specific immunosuppressants and targeted biological agents (11). With the rapid advancement of the NMOSD research field, the randomized controlled trials (RCT) of targeted biological agents in NMOSD have obtained positive results (12–14), which have been approved for AQP4-NMOSD, including eculizumab, inebilizumab, and satralizumab.

Bibliometric analysis has been recently used as a quantitative analysis method for scientific research evolution (15). In addition, it can be used to identify hotspots and emerging trends in a specific field (16). With the evolution and broadening of the concept of NMOSD and the accumulated new data, there have been a growing number of publications about NMOSD. However, as far as we know, there is no bibliometric analysis on this topic. Our research aims to provide an overview of NMOSD over the past two decades with the purpose of assisting researchers, especially beginners, in understanding the field more quickly and effectively.

## Methods

### Data source and search strategy

**Figure 1:**
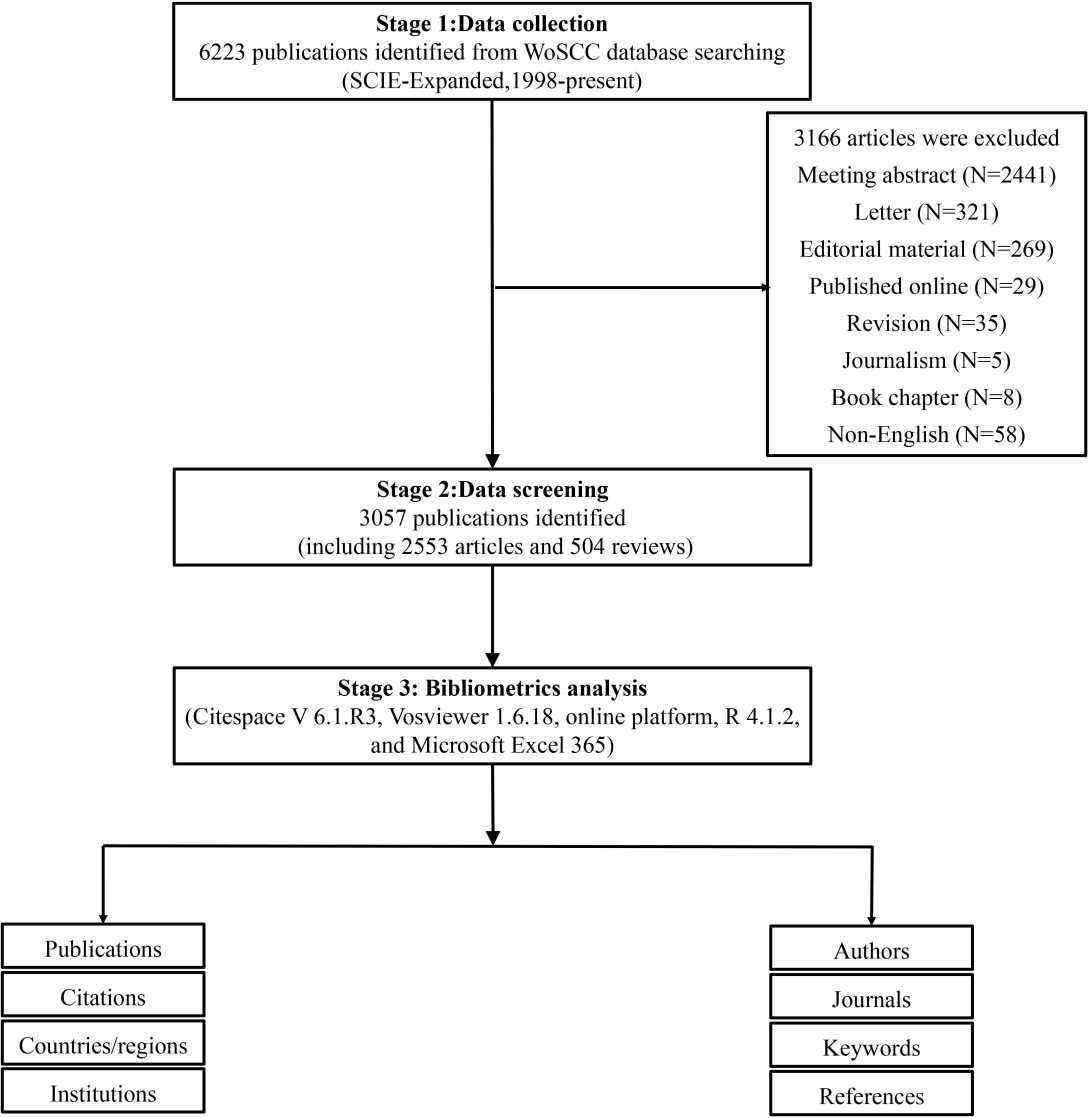
Flowchart of the literature search and screening in the study.

In the present study, we chose publications indexed in the Web of Science Core Collection (WoSCC) database, which is one of the most common databases used for bibliometric analysis (17). WoSCC, originating from Clarivate Analytics, has more than 12,000 international academic journals and is one of the most authoritative and comprehensive databases (18). The search strategy was as follows: TI = (neuromyelitis optica spectrum disorders OR neuromyelitis optica spectrum disorder OR neuromyelitis optica spectrum disease OR neuromyelitis optica spectrum diseases OR optica neuromyelitis spectrum disease OR neuromyelitis optica OR devic disease OR NMO OR NMOSD) OR AK = (neuromyelitis optica spectrum disorders OR neuromyelitis optica spectrum disorder OR neuromyelitis optica spectrum disease OR neuromyelitis optica spectrum diseases OR optica neuromyelitis spectrum disease OR devic disease OR NMO OR NMOSD). The retrieval time was from 1 January 2022 to 13 October 2022. At the same time, we limited the document types to “article” and “review”. We restricted the search to the English language. The flowchart for the selection of publications is shown in **Figure 1**.

### Data extraction and analysis

The data were extracted independently by two authors (YS and ZR). Full records and cited references of all the documents in the WoSCC were downloaded in txt or BibTeX format and then imported to CiteSpace 6.1.R3, 64 bits (Drexel University, Philadelphia, PA, USA), Microsoft Excel 365, VOSviewer 1.6.18 (Leiden University, The Netherlands), or R (Version 4.0.2), according to the software required for data analysis and visualization. CiteSpace was used to analyze the strongest citation bursts of references and keywords, investigate the research status, identify hotspots, and determine the development trend (19). VOSviewer was used to analyze the collaborative networks between countries, institutions, journals, and authors, reference co-citation, and keyword overlay visualization. Microsoft Excel 365 was used to analyze the trends of annual publications and the citations of the publications. The Biblioshiny packages in R were used to conduct collaboration network analysis among countries and the annual change pattern of journals. The https://bibliometric.com/ was used to analyze the changing trend of the annual publication quantity in the top 10 countries/regions and the geographic distribution map of different countries/regions. Each node represents a different parameter, including country, institution, keyword, etc. The parameter weight determines the size of the node. The heavier the weight, the bigger the node. Nodes and lines are colored according to the cluster they belong to. The distance between any two circles indicates the relatedness of their co-authorship and co-citation links, and the thickness of the connecting line indicates the strength of the link.

### Research ethics

Ethical approval was not required because no patients or animals were included in this study.

## Results

### The annual growth trend of publications

**Figure 2:**
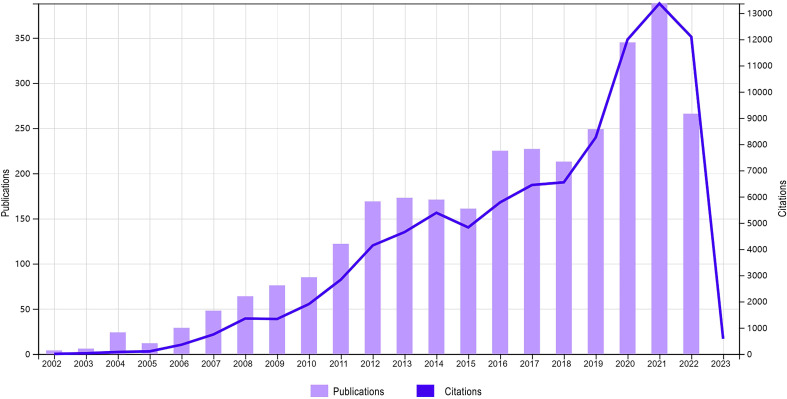
The annual publications and citations of NMOSD articles from 2002 to 2022.

A total of 3,057 publications on NMOSD, including 2,553 articles and 504 reviews, were retrieved from the WOSCC database on 13 October 2022. It was found that the overall trend of the number of published articles significantly increased from 2002 to 2022, especially in the last two years, indicating that the research on NMOSD has increasingly attracted scholars’ attention. The number of publications reached a peak in 2021, with a total of 388 articles and 13,349 citations (**Figure 2**). The total number of citations and the number of citations after the removal of self-citations were 89,131 and 39,875, respectively, and the average citation frequency of the publications was 29.19 times. The H index of the academic field was 124 from 2002 to 2022.

### Distribution of countries/regions and institutions

**Figure 3:**
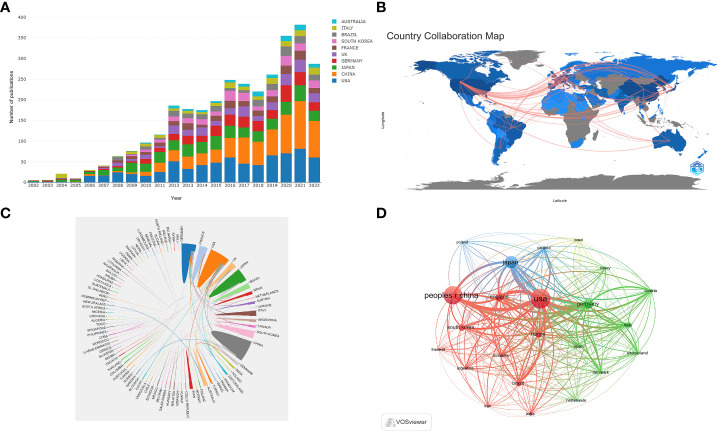
**(A)** The changing trend of the annual publication quantity in the top 10 countries/regions from 2002 to 2022; **(B)** Geographic distribution map of different countries/regions in the NMOSD field; **(C)** The international collaboration visualization map of countries/regions; **(D)** Network visualization showing the relationship between countries.

**Figure 4:**
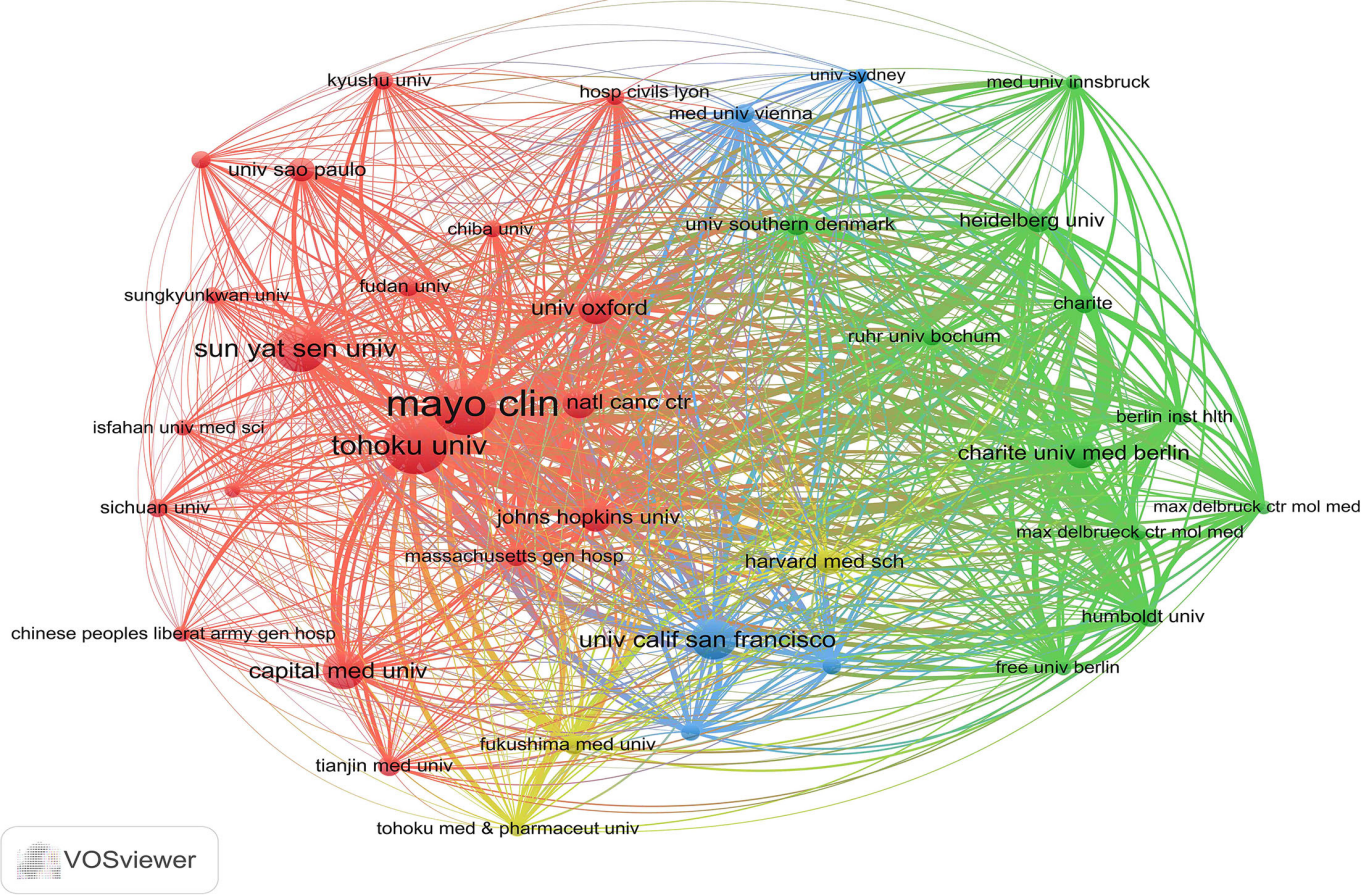
The network visualization of the citation analysis of the institutions.

**Table 1** shows the top 10 most prolific countries and institutions in the field related to NMOSD, along with their number of publications (NP), the total number of citations (NC), the average number of citations (AC), and the H-index. Among these countries, the United States (USA) published the most papers (n = 728, 23.81%) and had the most citations (31,808), followed by China (n = 663, 21.69%) and Japan (n = 415, 13.58%). It is noteworthy that of the top 10 productive countries, the NC and AC of China were relatively low even though China ranks second in the NP. **Figure 3A** shows the annual number of publications of the top 10 most productive countries/regions, showing that the number of publications in the field of NMOSD has grown fast. To study the cooperation between different countries, we used the Biblioshiny packages to analyze the data and conduct visualizations. **Figure 3B** shows that the map of cooperation among different countries/regions is complex. As shown in **Figure 3C**, the international collaboration map among countries/regions indicates that the USA collaborated most closely with Germany and China. The citation relationship between countries was analyzed by VOSviewer (**Figure 3D**). Only countries/regions with a minimum number of 30 publications were included. Of the 24 countries and regions that met this threshold, the top 5 with the largest TLS ranked as follows: the USA (Total Link Strength = 42,257), Germany (TLS = 26,351), Japan (TLS = 22,574), China (TLS = 21,679), and England (TLS = 19,326). **Table 1** summarizes the top 10 most influential institutions. The institutions with the highest NP values were the Mayo Clinic (NP = 167), followed by Tohoku University (NP = 164), and the University of California System (NP = 148). The most significant AC scores were from the Mayo Clinic (AC = 125.52), Tohoku University (AC = 78.3), and the University of Oxford (AC = 77.18). The distribution of the top 10 institutions was as follows: three institutions in the United States and three institutions in Germany. The citation relationship between institutions was analyzed by VOSviewer (**Figure 4**). Only countries/regions with a minimum number of 30 publications were included. Of the 39 countries/regions that met this threshold, the top 5 with the largest TLS ranked as follows: the Mayo Clinic (TLS = 11,804), Tohoku University (TLS = 8,913), Charité Berlin University of Medicine (TLS = 7,114), Oxford University (TLS = 6,056), and Heidelberg University (TLS = 5,542).

**Table 1:** The top 10 countries/regions and institutions contributing to neuromyelitis optica spectrum disorder research.

Rank	Country	Number of publications	Number of citations	Average Number of Citations	H-Index	Institutions	Number of publications	Number of citations	Average Number of Citations	Location
1	USA	728	31808	52.94	89	Mayo Clinic	167	19695	125.52	USA
2	China	663	5586	11.06	38	Tohoku University	164	12109	78.3	Japan
3	Japan	415	17882	48.51	67	University of California System	148	6394	48.46	USA
4	Germany	303	16162	62.09	65	Charite Universitatsmedizin Berlin	131	7883	68.81	Germany
5	England	245	14070	61.56	63	Free University of Berlin	131	7883	68.81	Germany
6	France	202	11140	58.64	53	Humboldt University of Berlin	131	7883	68.81	Germany
7	Republic of Korea	197	5231	28.79	41	Sun Yat Sen University	126	1682	14.67	China
8	Brazil	168	5614	35.8	32	University of Oxford	121	8921	77.18	England
9	Italy	154	6153	42.38	42	University of California San Francisco	113	5893	57.97	USA
10	Australia	93	5078	56.35	28	Udice French Research Universities	110	6479	61.11	France

### Authors and co-cited authors

**Figure 5:**
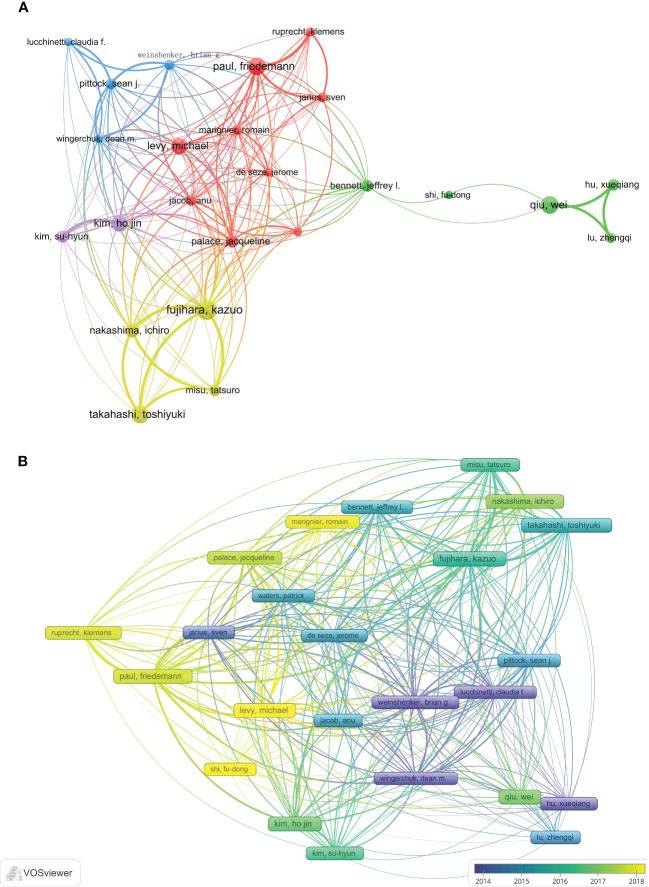
**(A)** The network visualization of the co-authorship analysis of the authors. **(B)** The overlay visualization map of author citation analysis based on VOSviewer. The purple nodes represent the authors that participated in the early research in this field, while the yellow nodes reflect the authors who engaged in later research.

A total of 200 authors participated in the publication of NMOSD articles. **Table 2** shows the top 10 most productive authors and co-cited authors who contributed to NMOSD. Fujihara, K was the most productive author, with 117 articles and 8,573 citations. The author with the most citations was Weinshenker, BG (16,250 citations), followed by Pittock, SJ (11,068 citations). Wingerchuk, DM, was the most co-cited author, with 2,549 citations, and he also had the highest centrality (0.04). The minimum number of author publications was set at 35, and 24 authors were selected for author co-authorship analysis using the VOSviewer. The co-authorship analysis (**Figure 5A**) showed that the authors were divided into five clusters. The red cluster (nine authors) was the largest co-authorship cluster. We also performed the author citation analysis using the VOSviewer software. The overlay visualization (**Figure 5B**) shows that the authors Lucchinetti, CF, Weinshenker, BG, Wingerchuk, DM, and Hu Xueqiang appeared were connected with the studies published during the earlier years of the range we used, while Levy, Michael, Marignier, Romain and Shi Fudong were the emerging authors in the studies published in recent years. The top three authors with the largest TLS were Fujihara, K (TLS = 4732), Paul, F (TLS = 4317), and Weinshenker, BG (TLS = 3483).

**Table 2:** The top 10 most productive authors and co-cited authors who contributed to neuromyelitis optica spectrum disorder research.

Rank	Author	Number of publications	Number of citations	Average Number of Citations	H-index	Co-cited Author	Number of citations	Centrality
1	Fujihara, K	117	8573	77.02	46	Wingerchuk, DM	2549	0.04
2	Paul, F	97	7128	82.06	45	Lennon, VA	1478	0.01
3	Kim, HJ	96	4028	44.46	36	Jarius, S	1186	0.07
4	Jarius, S	91	9052	108.44	44	Pittock, SJ	1008	0.01
5	Takahashi, T	84	3018	38.06	27	Lucchinetti, CF	665	0.01
6	Palace, J	76	5629	76.97	36	Weinshenker, BG	619	0.02
7	Weinshenker, BG	73	16250	228.05	47	Misu, T	531	0.03
8	Pittock, SJ	69	11068	164.78	41	Polman, CH	524	0.00
9	Misu, T	68	4871	74.94	38	Kitley, J	514	0.02
10	Nakashima, I	66	7014	109.06	39	Kim, SH	509	0.06

### Journals and co-cited journals

**Figure 6:**
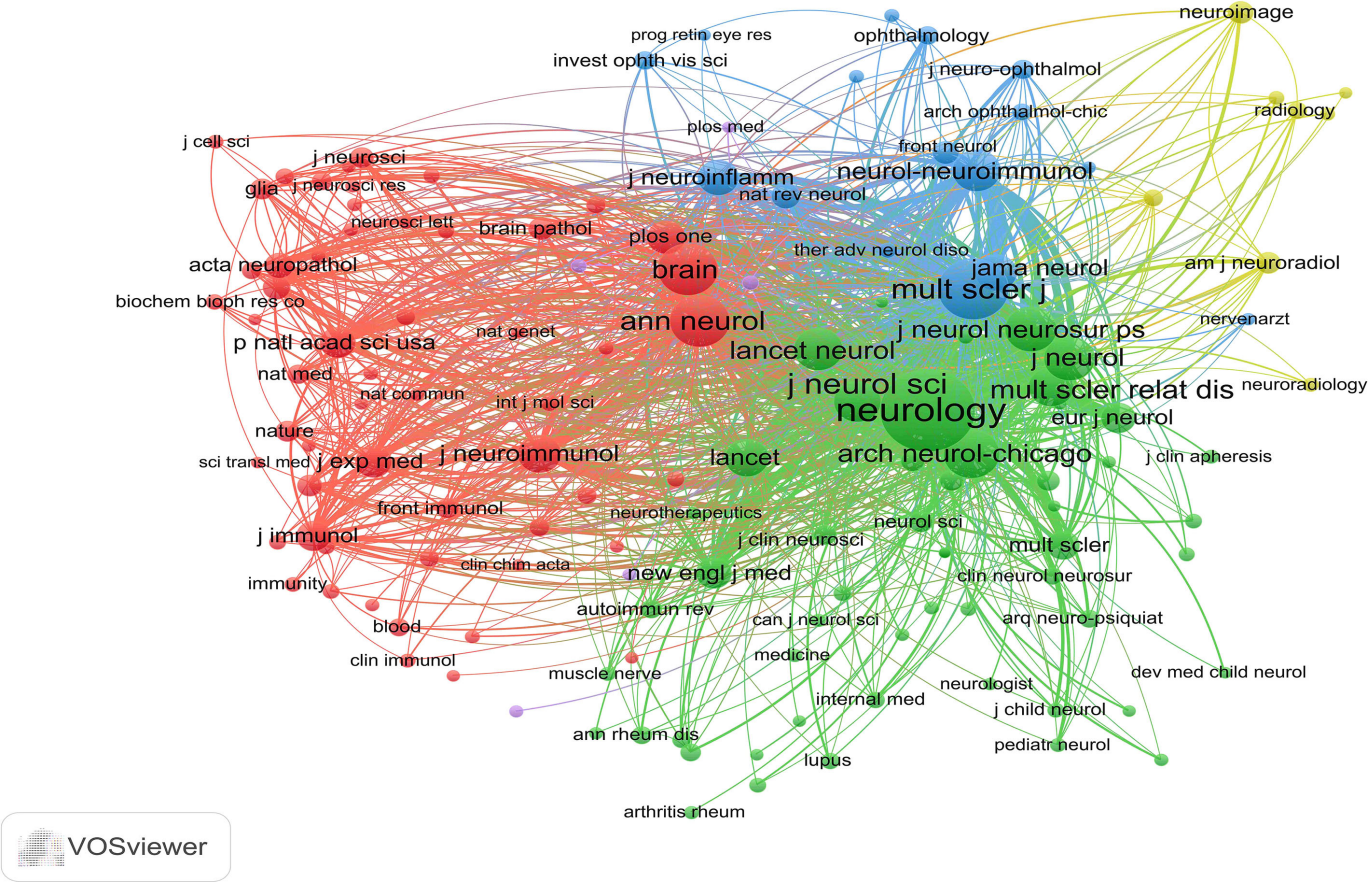
The network visualization of the co-citation analysis of the journals.

**Figure 7:**
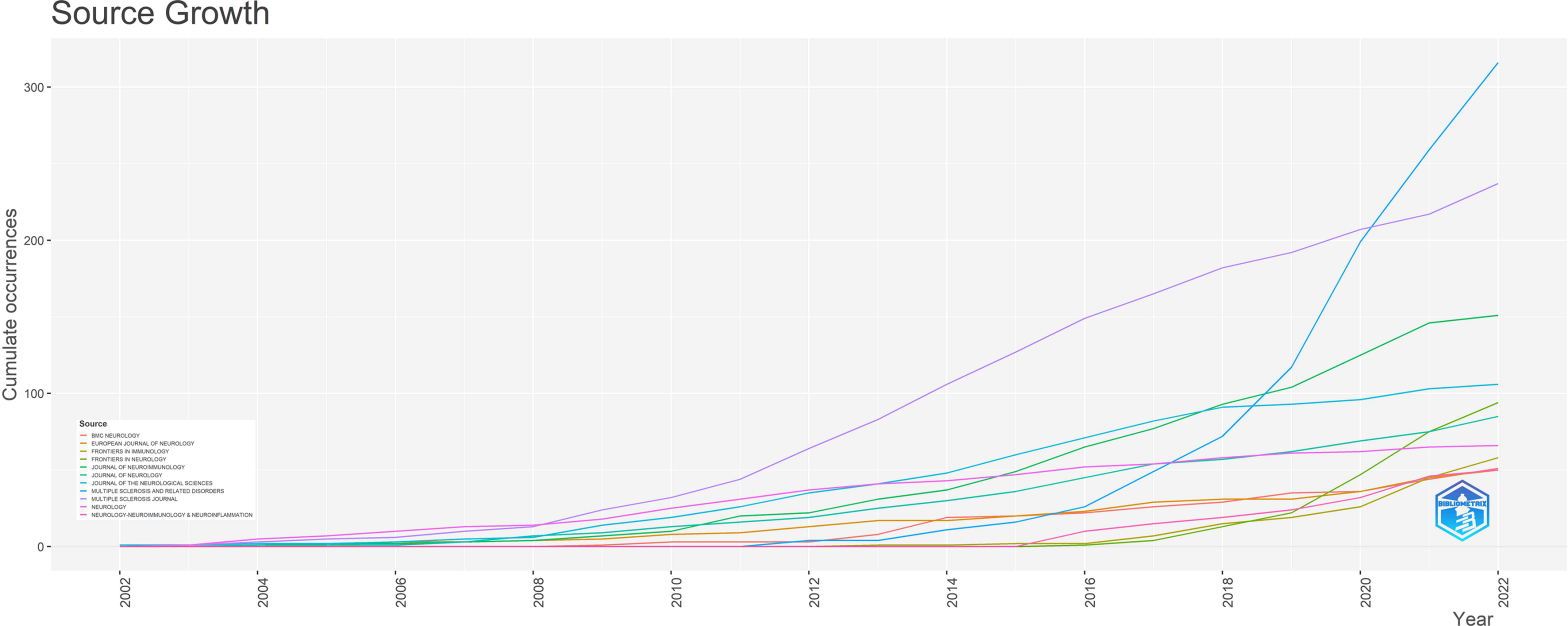
The annual change pattern of journal frequency.

A total of 3,057 NMOSD-related publications were published in 198 journals. **Table 3** shows the top 10 journals and the top 10 journals with co-citations. The journal with the most publications was *Multiple Sclerosis and Related Disorders* (n = 316, 10.34%), followed by *Multiple Sclerosis Journal* (n = 240, 7.85%), and the *Journal of Neuroimmunology* (n = 151, 4.94%). It can also be seen from **Table 3** that *Neurology* (2,853 co-citations) was the most co-cited journal, followed by *Multiple Sclerosis Journal* (2,144 co-citations), and *Annals of Neurology* (1,912 co-citations). Furthermore, 10 journals were co-cited more than 1,000 times, and 6 journals had an impact factor (IF) greater than 10. The *Lancet* had the highest IF among the top 10 co-cited journals. **Figure 6** demonstrates the journal co-citation analysis by VOSviewer software. Only journals with a minimum of 100 publications were included, and 137 journals were selected for analysis. The top three journals with the largest TLS were *Neurology* (TLS = 601882), *Multiple Sclerosis Journal* (TLS = 374029), and *Annals of Neurology* (TLS = 246728). The change pattern in the annual occurrence frequency of journals is shown in **Figure 7**. The number of publications in *Multiple Sclerosis and Related Disorders* increased rapidly from 2008 to 2022. The number of publications in the Multiple Sclerosis Journal increased steadily from 2008 to 2022.

**Table 3:** The top 10 most productive journals and co-cited journals for neuromyelitis optica spectrum disorder research.

Rank	Journals	Number of publications	Number of citations	Average number of citations	IF and JCR division (2021)	Co-cited journals	Number of co-citations	IF and JCR division (2021)	Centrality
1	*Multiple Sclerosis and Related Disorders*	316	2403	8.83	4.808, Q2	*Neurology*	2853	11.8, Q1	0.01
2	*Multiple Sclerosis Journal*	240	8525	36.83	5.855, Q1	*Multiple Sclerosis Journal*	2144	5.855, Q1	0.01
3	*Journal of Neuroimmunology*	151	985	34.38	3.221, Q3	*Annals of Neurology*	1912	11.274, Q1	0.01
4	*Journal of the Neurological Sciences*	106	2616	25.52	4.553, Q2	*Brain*	1840	15.255, Q1	0.01
5	*Frontiers in Neurology*	94	788	9	4.086, Q2	*Archives of Neurology*	1680	7.419, Q1	0.01
6	*Journal of Neurology*	86	3082	36.94	6.682, Q1	*Lancet Neurology*	1636	59.935, Q1	0.02
7	*Neurology*	67	10866	164.93	11.8, Q1	*Journal of Neurology Neurosurgery and Psychiatry*	1601	13.654, Q1	0.01
8	*Frontiers in Immunology*	58	556	9.88	8.786, Q1	*Lancet*	1555	202.731, Q2	0.01
9	*BMC Neurology*	52	663	13.02	2.903, Q3	*Journal of Neurology*	1466	6.682, Q1	0.01
10	*European Journal of Neurology*	52	1477	28.79	6.288, Q1	*Journal of the Neurological Sciences*	1382	4.553, Q2	0.01

### Co-cited references and references burst

**Figure 8:**
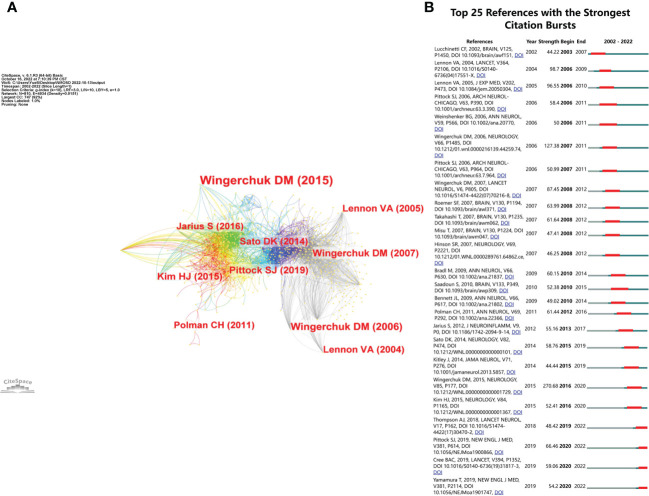
**(A)** The visualization of co-cited references in the NMOSD field. **(B)** The top 25 references with the strongest citation bursts.

We summarized the top 10 most cited articles in the NMOSD field in **Table 4**. All of them were published between 2002 and 2015, including international guidelines and diagnostic criteria for NMOSD (9), humoral mechanisms and biomarkers (AQP4 antibody) for NMOSD (5, 7), and clinical trials for NMOSD (20). The reference co-cited analysis was performed by CiteSpace software. **Figure 8A** shows the first author and 10 most-cited references. The color of the link between the two circles represents the year of the first co-citation of the two references. The references with citation bursts are those that have been cited more frequently over time. We listed the top 25 references with the strongest citation bursts in **Figure 8B**. The reference with the strongest burstiness (strength = 270.68) was the article entitled “International consensus diagnostic criteria for neuromyelitis optica spectrum disorders”, which was published in *Neurology* by the International Panel for NMO Diagnosis (IPND) in 2015 (9). There were four articles still burst until 2022 that mainly involved targeted immunotherapy in NMOSD, including eculizumab, inbelizumab, and satralizumab (12, 13, 21).

**Table 4:** The top 10 cited references for neuromyelitis optica spectrum disorder research.

Rank	Title	Year	Author	Journal	Number of Co-citations
1	International consensus diagnostic criteria for neuromyelitis optica spectrum disorders	2015	Wingerchuk, DM	*Neurology*	2180
2	A serum autoantibody marker of neuromyelitis optica: distinction from multiple sclerosis	2004	Lennon, VA	*Lancet*	2154
3	Revised diagnostic criteria for neuromyelitis optica	2006	Wingerchuk, DM	*Neurology*	1957
4	The spectrum of neuromyelitis optica	2007	Wingerchuk, DM	*Lancet Neurology*	1497
5	A role for humoral mechanisms in the pathogenesis of Devic’s neuromyelitis optica	2002	Lucchinetti, CF	*Brain*	862
6	International Pediatric Multiple Sclerosis Study Group criteria for pediatric multiple sclerosis and immune-mediated central nervous system demyelinating disorders: revisions to the 2007 definitions	2013	Krupp, LB	*Multiple Sclerosis Journal*	586
7	Pattern-specific loss of aquaporin-4 immunoreactivity distinguishes neuromyelitis optica from multiple sclerosis	2007	Roemer, SF	*Brain*	515
8	Brain abnormalities in neuromyelitis optica	2006	Pittock, SJ	*Archives of Neurology*	504
9	Neuromyelitis optica brain lesions localized at sites of high aquaporin 4 expression	2006	Pittock, SJ	*Archives of Neurology*	491
10	Contrasting disease patterns in seropositive and seronegative neuromyelitis optica: A multicentre study of 175 patients	2012	Jarius, S	*Journal of Neuroimmunology*	471

### Keywords co-occurrence and burst analysis

**Figure 9:**
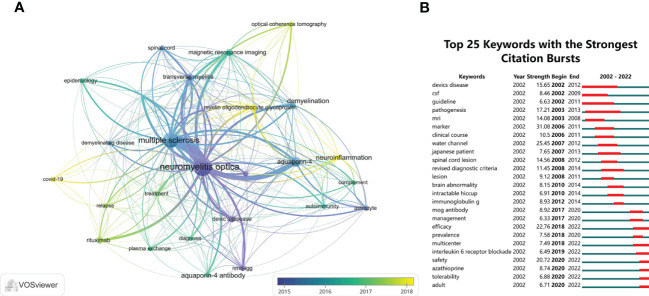
**(A)** The overlay visualization map of the co-occurrence keywords performed by VOSviewer software. **(B)** The top 25 keywords with the strongest citation bursts of publications in the field of NMOSD from 2002 to 2022.

We used VOSviewer software to perform the keywords co-occurrence analysis. In total, 24 keywords with a minimum of 35 occurrences were extracted after merging keywords with the same meaning. An overlay visualization map of keywords is shown in **Figure 9A**, which indicates the changes in the keywords over time. The purple nodes indicate early hotspots and the yellow nodes indicate emerging hotspots. The keywords gradually changed from “transverse myelitis”, “nmo-igg”, “astrocyte”, “demyelination”, and “aquaporin-4” to “relapse”, “treatment”, “rituximab”, “plasma exchange”, “diagnosis”, and “magnetic resonance imaging”. It was seen that “COVID-19”, “neuroinflammation”, and “myelin oligodendrocyte glycoprotein” were keywords that frequently appeared more recently, suggesting that they will be future research hotspots. **Figure 9B** shows the top 25 keywords with the strongest citation bursts, which were conducted by CiteSpace software. The keyword “marker” had the strongest burst (strength = 31.08) and burst from 2006 to 2011; the terms “devics disease” (beginning in 2002 and ending in 2012) and “pathogenesis” (beginning in 2003 and ending in 2013) had the longest burst time. Whereas “efficacy”, “multicenter”, “interleukin 6 receptor blockade”, “safety”, “azathioprine”, “tolerability”, and “adult” were still burst.

## Discussion

In the past two decades, the number of annual publications and citations in the NMOSD field has increased steadily and reached a peak in 2021. With the discovery of the AQP4 antibody, researchers have put in great efforts and there have been great strides made in the diagnosis and treatment of NMOSD. Therefore, we conducted a bibliometric analysis that provided a comprehensive overview of the development of NMOSD research and predicts future research hotspots. This is the first bibliometric analysis of NMOSD. As of 13 October 2022, 3,057 publications had been published in 198 journals by 200 authors from 200 institutions in 93 countries/regions.

It has long been controversial whether NMOSD is an independent disease or a subgroup of MS. The discovery of the anti-NMO antibody in 2004 and its target AQP4 in 2005 revolutionized the diagnosis and treatment of the disease and provided insight into its pathophysiology (5, 6). The discovery of the AQP4 antibody facilitated early diagnosis and the use of immunotherapy to prevent attacks and improve outcomes. A better understanding of the pathogenesis of NMOSD has led to the rational development of mechanism-based therapies. Since then, the NMOSD field has rapidly developed and shown continued growth. The number of NMOSD publications and citations reached a new level in 2020 and continues to grow until this day. The possible reason for this phenomenon is that RCTs on many targeted immunotherapies for NMOSD have been published in high-quality journals (13, 14, 22), and, based on clinical trial results published over the past three years, some of these biological agents have been approved for the treatment of NMOSD. The success of these studies has greatly promoted the progress of NMOSD treatment.

In terms of country analysis, the USA contributed the most NP, NC, AC, and H-index, suggesting that the USA is a highly productive and leading country in the NMOSD field. The USA has the most outstanding researchers and institutions globally, suggesting the USA holds the leading position in the NMOSD field. Although the NP of China ranked second, the numbers of NC, AC, and H-index were relatively low.

Based on these data, it appears that China has an imbalance between quantity and quality. This issue may be addressed in the following aspects: (1) enhancing collaboration with other countries, especially the USA, Japan, and Germany; and (2) keeping a close eye on scientific innovation and conducting more in-depth studies to improve publication quality. Among the top 10 most productive institutions, 60% were from the USA and Germany. This might explain why the USA was the most productive country in the NMOSD field. These findings indicated that global academic resources are unbalanced and that the establishment of world-class academic institutions is an important basis for improving national academic status.

Of the top 10 prolific authors, Fujihara, K was the most productive author with 117 articles and 8,573 citations in the NMOSD field, while the NC and AC of Weinshenker, BG were ranked first. Furthermore, among the top co-cited authors, it is evident that Wingerchuk, DM had the highest centrality. Fujihara, K, from Fukushima Medical University School of Medicine, participated in many clinical trials of NMOSD (12, 13, 23) and was involved in the development of guidelines for demyelinating diseases of the central nervous system, including NMOSD, MS, and myelin oligodendrocyte glycoprotein antibody-associated disease (9, 24, 25). Weinshenker, BG is a neurologist from the Mayo Clinic. He is famous for his contributions to exploring the diagnosis and treatment of NMOSD. He, as the corresponding author, published the article about the serum autoantibody marker of neuromyelitis optica (5); since then, NMOSD has been recognized as an independent disease different from MS. Wingerchuk, DM is also from Mayo Clinic, and had many collaborations with Weinshenker, BG in the NMOSD field (26–28); he, as first author, published the international consensus diagnostic criteria for NMOSD (9), and also published many high-quality reviews in the *Lancet* and *The New England Journal of Medicine (* 1, 27). The VOSviewer software automatically divided all authors into different clusters. The authors from the same clusters contributed excellent scientific works to their region. We also observed Bennett JL as a bridge connecting Shi Fudong and other authors from different institutions (**Figure 4A**). They jointly published an open-label, multicenter, randomized, phase 2 trial of NMOSD (22). Collaboration has always been an important requirement for the advancement of scientific discovery.

In the top 10 most productive journals, *Multiple Sclerosis and Related Disorders* and *Multiple Sclerosis Journal* were the most productive journals, which were the specialist journals in the MS and NMOSD fields. In the future, it is expected that more important research results will be published in this journal, and the impact factor and scientific value will improve. The other remaining journals specialized in Neurology and Immunology. It is important for scientists to follow these journals to see how NMOSD is progressing and what might happen in the future. Furthermore, the journal analysis could help researchers avoid delays in the research process by quickly identifying the best journals for submission.

Co-citation analysis is an effective method to assess the level of correlation between articles. It is generally considered that the more frequently an article is cited, the greater its importance in the professional field (29). Most of the top 10 cited references were published in top-ranked journals, and three articles were written by Wingerchuk, DM (9, 27, 30); this result also confirmed the results of the author analysis. In the top 10 cited references, three of them were the diagnostic criteria for NMOSD. The formulation of guidelines plays an important role in the treatment of diseases. As a clinical decision-making tool to narrow the gap between current evidence-based medicine and clinical practice, clinical guidelines could improve diagnosis and treatment levels, standardize medical behavior, and improve service quality. Three of the top 10 cited references described the serological mechanism of NMOSD and the relationship between anti-AQP4 and NMOSD. The second cited article, entitled “A serum autoantibody marker of neuromyelitis optica: distinction from multiple sclerosis”, identified an NMO-IgG (5), which is a specific marker autoantibody that binds at or near the blood-brain barrier. It distinguishes NMO from MS. This important finding broadened the pathological concept of NMOSD. Additionally, the 10 most cited articles (20) were published in the *Journal of Neuroinflammation*”, which provided an overview of the clinical features of NMOSD and demonstrated a few distinct characteristics in seropositive and seronegative patients. Since the discovery of anti-AQP4, most of the hot topics have focused on seropositive patients, while some seronegative patients with typical NMOSD performance have also gradually attracted the attention of researchers in recent years. Therefore, on the basis of understanding the clinical characteristics of seropositive patients, we should further explore the field of seronegative patients, which is conducive to the diagnosis of seronegative patients, precision treatment, and guidance of clinical research. From the reference burst, the first burst reference emerged in 2003 owing to one study published in 2002 (7). This article used the pathological method to investigate the importance of humoral mechanisms, including complement activation, in producing the necrotizing demyelination seen in the spinal cord and optic nerves. The strongest burst reference was the international consensus diagnostic criteria for NMOSD (9), which was the authoritative publication to guide clinicians in the NMOSD field. There were four articles still burst (12, 13, 21, 24), and three of them were RCTs on targeted immunotherapy of NMOSD. The successful publication of these articles provided evidence-based medical evidence for targeted immunotherapy for NMOSD. Among them, eculizumab, inbelizumab, and satralizumab have been approved for AQP4-NMOSD patients. These targeted biological agents are expected to improve the symptoms rapidly, accurately, and effectively, reduce the risk of relapse of NMOSD, reduce the side effects of steroids and traditional immunosuppressants, and reduce the disease burden on patients. There will need to be further exploration of more targeted biological agents for different targets in the future.

Keywords are a summary of the core ideas of an article and are generally considered to be important indicators reflecting research direction and hotspots in a specific field. The evolution and change of keywords over time reflect, to some extent, the development of hotspots and can guide future research directions. Through the overlay visualization, we can observe that the keywords gradually shifted from serological mechanisms and clinical characteristics to diagnosis and treatment for NMOSD, which also indicates that the research hotspots gradually changed from the basic study to clinical translation. The yellow nodes indicate emerging hotspots. We found that the research topics “COVID-19”, “neuroinflammation”, and “myelin oligodendrocyte glycoprotein” have received attention in recent years due to the COVID-19 pandemic, which has been a challenge for patients with autoimmune diseases. For patients with NMOSD, long-term use of steroids or traditional immunosuppressants may cause an immunosuppressive state. Therefore, they were at high risk of COVID-19 infection, and infection with COVID-19 may lead to NMOSD relapse or aggravation. Furthermore, the COVID-19 vaccination was an additional concern. On the one hand, it was necessary to consider whether the COVID-19 vaccination would lead to a relapse of NMOSD (31, 32). On the other hand, some patients would have new-onset NMOSD after the COVID-19 vaccination (33–36). This is why COVID-19 became a hot topic in NMOSD research. Since the discovery of anti-AQP4 in 2005 (6), the focus of research has always been on patients with anti-AQP4 seropositive NMOSD. In anti-AQP4 seronegative patients, anti-myelin oligodendrocyte glycoprotein (MOG) has been detected in patients with optic neuritis and longitudinally extensive transverse myelitis. MOG antibody-associated disease (MOGAD) has the same clinical characteristics as demyelinating diseases of the central nervous system, such as NMOSD and MS, but the difference is that its main manifestation is a monophasic or relapsing disease course, which has no marked sex or racial predominance in populations with MOGAD. The histopathological characteristics, imaging characteristics, treatment response, and outcomes of MOGAD are different from those of MS and NMOSD, so it is necessary to establish its diagnostic criteria as a distinct entity. The researchers still need to explore this area in the future.

The word “marker” was the strongest burst keyword, because in the development of NMOSD, the discovery of anti-AQP4 is very important. After the discovery of anti-AQP4 as a serum marker, NMOSD was separated from MS as an independent disease (6). Since then, the number of publications on NMOSD has increased over the past year, and many articles (37–40) have been published on its epidemiology, diagnosis, clinical characteristics, treatment regimens, treatment response, and outcomes. “Pathogenesis” was the keyword that burst for the longest time. This was because after exploring and establishing the pathogenesis of NMOSD, we could better understand its diagnosis and treatment and develop targeted biological agents acting on different targets of its pathogenesis. It can be seen from the keyword burst analysis that “safety”, “efficacy”, “tolerability”, and “multicenter” were the keywords around RCT, which indicates that the research of NMOSD entered the era of evidence-based medicine. The “interleukin 6 receptor blockade” was still burst, which due to the RCT on satralizumab showed positive results. Tocilizumab, another targeting IL-6R drug, also carried out a multi-center, randomized, phase II trial in NMOSD (22), which compared the safety and efficacy of tocilizumab and azathioprine in patients with highly relapsing NMOSD. The results showed that tocilizumab significantly reduced the risk of a subsequent NMOSD relapse compared with azathioprine. Tocilizumab might therefore be another safe and effective treatment to prevent relapses in patients with NMOSD. It can be seen from the above mentioned keyword analysis that future research will focus on the targeted immunotherapy of NMOSD, on the development of targeted biological agents for different targets of its pathogenesis, and on conducting RCTs on the existing targeted biological agents to obtain higher-quality evidence-based medical evidence. Targeted immunotherapy will quickly and effectively reduce the relapse of NMOSD and further reduce the disease burden for patients.

## Limitations

This study has some limitations. First, this study utilized the WoSCC database as its data source and the data from other databases, such as PubMed, Cochrane, Scopus, and Embase library databases, has not been included. We used the same search strategy to search PubMed, and a total of 3,341 studies related to NMOSD were retrieved. After merging and duplicating the studies from WOS and PubMed, except for a few of them, almost all of the studies in PubMed were included in the WOS database, especially the significant influence and epoch-making significant studies in the field of NMOSD. Additionally, with the availability of many bibliometric indicators and containing 12,000 influential high-quality journals from countries worldwide, the WOS database is regarded as one of the most authoritative and optimal databases for the bibliometric analysis of scientific publications. Therefore, this limitation would not have impacted the analysis results. Second, only article/review publications in English were searched, and non-English or non-article/review publications were not included in this search, so some publications may have been missed. Third, the number of citations was not a complete indicator of the quality of publications because it takes time to cite manuscripts. Older publications may be cited more frequently, so influential manuscripts may take several years to be cited. Finally, bibliometric software such as CiteSpace and VOSviewer cannot provide statistical functions, so it is impossible to understand the actual situation of publications in different countries/regions.

## Conclusion

To our knowledge, this study is the first bibliometric analysis of publications in the field of NMOSD using visualization software to obtain the direction, hotspots, and developments of this field, which may provide helpful information for researchers. Future research hotspots should obtain higher-quality evidence-based medical evidence on targeted immunotherapy in the NMOSD field.

## Data availability statement

The raw data supporting the conclusions of this article will be made available by the authors, without undue reservation.

## Author contributions

TC and ZL contributed to the conception and design of the study. YS, ZR, and SL performed the statistical analysis. YS, ZR, and SL were involved in writing the manuscript and drafting the article. YS, ZR, and SL contributed to data acquisition. All authors contributed to the article and approved the submitted version.

## References

[1] Wingerchuk DM Lucchinetti CF. Neuromyelitis optica spectrum disorder. N Engl J Med (2022) 387(7):631–9. doi: 10.1056/NEJMra1904655 36070711

[2] Papp V Magyari M Aktas O Berger T Broadley SA Cabre P. Worldwide incidence and prevalence of neuromyelitis optica: a systematic review. Neurology (2021) 96(2):59–77. doi: 10.1212/wnl.0000000000011153 33310876 PMC7905781

[3] Wingerchuk DM Hogancamp WF O’Brien PC Weinshenker BG. The clinical course of neuromyelitis optica (Devic’s syndrome). Neurology (1999) 53(5):1107–14. doi: 10.1212/wnl.53.5.1107 10496275

[4] Jarius S Wildemann B. The history of neuromyelitis optica. part 2: ‘Spinal amaurosis’, or how it all began. J Neuroinflamm (2019) 16(1):280. doi: 10.1186/s12974-019-1594-1 PMC6935230 31883522

[5] Lennon VA Wingerchuk DM Kryzer TJ Pittock SJ Lucchinetti CF Fujihara K. A serum autoantibody marker of neuromyelitis optica: distinction from multiple sclerosis. Lancet (2004) 364(9451):2106–12. doi: 10.1016/s0140-6736(04)17551-x 15589308

[6] Lennon VA Kryzer TJ Pittock SJ Verkman AS Hinson SR. Igg marker of optic-spinal multiple sclerosis binds to the aquaporin-4 water channel. J Exp Med (2005) 202(4):473–7. doi: 10.1084/jem.20050304 PMC2212860 16087714

[7] Lucchinetti CF Mandler RN McGavern D Bruck W Gleich G Ransohoff RM. A role for humoral mechanisms in the pathogenesis of devic’s neuromyelitis optica. Brain (2002) 125(Pt 7):1450–61. doi: 10.1093/brain/awf151 PMC5444467 12076996

[8] Lucchinetti CF Guo Y Popescu BF Fujihara K Itoyama Y Misu T. The pathology of an autoimmune astrocytopathy: lessons learned from neuromyelitis optica. Brain Pathol (2014) 24(1):83–97. doi: 10.1111/bpa.12099 24345222 PMC3905574

[9] Wingerchuk DM Banwell B Bennett JL Cabre P Carroll W Chitnis T. International consensus diagnostic criteria for neuromyelitis optica spectrum disorders. Neurology (2015) 85(2):177–89. doi: 10.1212/wnl.0000000000001729 PMC4515040 26092914

[10] Kleiter I Gahlen A Borisow N Fischer K Wernecke KD Wegner B. Neuromyelitis optica: evaluation of 871 attacks and 1,153 treatment courses. Ann Neurol (2016) 79(2):206–16. doi: 10.1002/ana.24554 26537743

[11] Waliszewska-Prosół M Chojdak-Łukasiewicz J Budrewicz S Pokryszko-Dragan A. Neuromyelitis optica spectrum disorder treatment-current and future prospects. Int J Mol Sci (2021) 22(6):2801. doi: 10.3390/ijms22062801 33802046 PMC7998461

[12] Pittock SJ Berthele A Fujihara K Kim HJ Levy M Palace J. Eculizumab in aquaporin-4-Positive neuromyelitis optica spectrum disorder. N Engl J Med (2019) 381(7):614–25. doi: 10.1056/NEJMoa1900866 31050279

[13] Cree BAC Bennett JL Kim HJ Weinshenker BG Pittock SJ Wingerchuk DM. Inebilizumab for the treatment of neuromyelitis optica spectrum disorder (N-momentum): a double-blind, randomised placebo-controlled phase 2/3 trial. Lancet (2019) 394(10206):1352–63. doi: 10.1016/s0140-6736(19)31817-3 31495497

[14] Traboulsee A Greenberg BM Bennett JL Szczechowski L Fox E Shkrobot S. Safety and efficacy of satralizumab monotherapy in neuromyelitis optica spectrum disorder: a randomised, double-blind, multicentre, placebo-controlled phase 3 trial. Lancet Neurol (2020) 19(5):402–12. doi: 10.1016/s1474-4422(20)30078-8 PMC7935419 32333898

[15] Wu K Liu Y Liu L Peng Y Pang H Sun X. Emerging trends and research foci in tumor microenvironment of pancreatic cancer: a bibliometric and visualized study. Front Oncol (2022) 12:810774. doi: 10.3389/fonc.2022.810774 35515122 PMC9063039

[16] Wu H Zhou Y Xu L Tong L Wang Y Liu B. Mapping knowledge structure and research frontiers of ultrasound-induced blood-brain barrier opening: a scientometric study. Front Neurosci (2021) 15:706105. doi: 10.3389/fnins.2021.706105 34335175 PMC8316975

[17] You Y Li W Liu J Li X Fu Y Ma X. Bibliometric review to explore emerging high-intensity interval training in health promotion: a new century picture. Front Public Health (2021) 9:697633. doi: 10.3389/fpubh.2021.697633 34368063 PMC8342813

[18] Wu H Li Y Tong L Wang Y Sun Z. Worldwide research tendency and hotspots on hip fracture: a 20-year bibliometric analysis. Arch Osteoporos (2021) 16(1):73. doi: 10.1007/s11657-021-00929-2 33866438

[19] Chen C. Searching for intellectual turning points: progressive knowledge domain visualization. Proc Natl Acad Sci U.S.A. (2004) 101 Suppl 1(Suppl 1):5303–10. doi: 10.1073/pnas.0307513100 PMC387312 14724295

[20] Jarius S Ruprecht K Wildemann B Kuempfel T Ringelstein M Geis C. Contrasting disease patterns in seropositive and seronegative neuromyelitis optica: a multicentre study of 175 patients. J Neuroinflamm (2012) 9:14. doi: 10.1186/1742-2094-9-14 PMC3283476 22260418

[21] Yamamura T Kleiter I Fujihara K Palace J Greenberg B Zakrzewska-Pniewska B. Trial of satralizumab in neuromyelitis optica spectrum disorder. N Engl J Med (2019) 381(22):2114–24. doi: 10.1056/NEJMoa1901747 31774956

[22] Zhang C Zhang M Qiu W Ma H Zhang X Zhu Z. Safety and efficacy of tocilizumab versus azathioprine in highly relapsing neuromyelitis optica spectrum disorder (Tango): an open-label, multicentre, randomised, phase 2 trial. Lancet Neurol (2020) 19(5):391–401. doi: 10.1016/s1474-4422(20)30070-3 32333897 PMC7935423

[23] Wingerchuk DM Fujihara K Palace J Berthele A Levy M Kim HJ. Long-term safety and efficacy of eculizumab in aquaporin-4 igg-positive nmosd. Ann Neurol (2021) 89(6):1088–98. doi: 10.1002/ana.26049 PMC8248139 33586143

[24] Thompson AJ Banwell BL Barkhof F Carroll WM Coetzee T Comi G. Diagnosis of multiple sclerosis: 2017 revisions of the mcdonald criteria. Lancet Neurol (2018) 17(2):162–73. doi: 10.1016/s1474-4422(17)30470-2 29275977

[25] Banwell B Bennett JL Marignier R Kim HJ Brilot F Flanagan EP. Diagnosis of myelin oligodendrocyte glycoprotein antibody-associated disease: international mogad panel proposed criteria. Lancet Neurol (2023) 22(3):268–82. doi: 10.1016/s1474-4422(22)00431-8 36706773

[26] Weinshenker BG Wingerchuk DM. Neuromyelitis spectrum disorders. Mayo Clin Proc (2017) 92(4):663–79. doi: 10.1016/j.mayocp.2016.12.014 28385199

[27] Wingerchuk DM Lennon VA Lucchinetti CF Pittock SJ Weinshenker BG. The spectrum of neuromyelitis optica. Lancet Neurol (2007) 6(9):805–15. doi: 10.1016/s1474-4422(07)70216-8 17706564

[28] Zalewski NL Morris PP Weinshenker BG Lucchinetti CF Guo Y Pittock SJ. Ring-enhancing spinal cord lesions in neuromyelitis optica spectrum disorders. J Neurol Neurosurg Psychiatry (2017) 88(3):218–25. doi: 10.1136/jnnp-2016-314738 27913626

[29] Wu H Tong L Wang Y Yan H Sun Z. Bibliometric analysis of global research trends on ultrasound microbubble: a quickly developing field. Front Pharmacol (2021) 12:646626. doi: 10.3389/fphar.2021.646626 33967783 PMC8101552

[30] Wingerchuk DM Lennon VA Pittock SJ Lucchinetti CF Weinshenker BG. Revised diagnostic criteria for neuromyelitis optica. Neurology (2006) 66(10):1485–9. doi: 10.1212/01.wnl.0000216139.44259.74 16717206

[31] Kong L Wang X Chen H Shi Z Lang Y Zhang Y. Relapses after sars-Cov-2 vaccination in patients with neuromyelitis optica spectrum disorder and multiple sclerosis. Mult Scler Relat Disord (2022) 68:104167. doi: 10.1016/j.msard.2022.104167 36170773 PMC9472679

[32] Stastna D Menkyova I Drahota J Hrnciarova T Kubala Havrdova E Vachova M. To be or not to be vaccinated: the risk of Ms or nmosd relapse after covid-19 vaccination and infection. Mult Scler Relat Disord (2022) 65:104014. doi: 10.1016/j.msard.2022.104014 35803085 PMC9250417

[33] Lévi-Strauss J Provost C Wane N Jacquemont T Mélé N. Nmosd typical brain lesions after covid-19 mrna vaccination. J Neurol (2022) 269(10):5213–5. doi: 10.1007/s00415-022-11229-1 PMC9214460 35731277

[34] Anamnart C Tisavipat N Owattanapanich W Apiwattanakul M Savangned P Prayoonwiwat N. Newly diagnosed neuromyelitis optica spectrum disorders following vaccination: case report and systematic review. Mult Scler Relat Disord (2022) 58:103414. doi: 10.1016/j.msard.2021.103414 35216789

[35] Francis AG Elhadd K Camera V Ferreira Dos Santos M Rocchi C Adib-Samii P. Acute inflammatory diseases of the central nervous system after sars-Cov-2 vaccination. Neurol Neuroimmunol Neuroinflamm (2023) 10(1):e200063. doi: 10.1212/nxi.0000000000200063 36411077 PMC9679888

[36] Motahharynia A Naghavi S Shaygannejad V Adibi I. Fulminant neuromyelitis optica spectrum disorder (Nmosd) following covid-19 vaccination: a need for reconsideration? Mult Scler Relat Disord (2022) 66:104035. doi: 10.1016/j.msard.2022.104035 35858498 PMC9254455

[37] Pittock SJ Zekeridou A Weinshenker BG. Hope for patients with neuromyelitis optica spectrum disorders - from mechanisms to trials. Nat Rev Neurol (2021) 17(12):759–73. doi: 10.1038/s41582-021-00568-8 34711906

[38] Bukhari W Clarke L O’Gorman C Khalilidehkordi E Arnett S Prain KM. The clinical profile of nmosd in Australia and new Zealand. J Neurol (2020) 267(5):1431–43. doi: 10.1007/s00415-020-09716-4 32006158

[39] Bukhari W Prain KM Waters P Woodhall M O’Gorman CM Clarke L. Incidence and prevalence of nmosd in Australia and new Zealand. J Neurol Neurosurg Psychiatry (2017) 88(8):632–8. doi: 10.1136/jnnp-2016-314839 28550069

[40] Carta S Le Duy D Rogemond V Derache N Chaumont H Fromont A. Anti-argonaute antibodies as a potential biomarker in nmosd. J Neurol Neurosurg Psychiatry (2023). doi: 10.1136/jnnp-2022-330707 36810322

